# Stress shielding following stemless anatomic total shoulder arthroplasty

**DOI:** 10.1177/17585732211058804

**Published:** 2021-11-24

**Authors:** William R Aibinder, Fares Uddin, Ryan T Bicknell, Ryan Krupp, Markus Scheibel, George S Athwal

**Affiliations:** 1 Department of Orthopaedic Surgery & Rehabilitation Medicine, 12298 SUNY Downstate Health Sciences University, Brooklyn, NY, USA; 2 Bahrain Royal Guard, 213439 Bahrain Defense Force, Riffa, Bahrain; 3 Department of Surgery and Mechanical and Materials Engineering, Human Mobility Research Centre, 4257 Queen's University, Kingston, ON, Canada; 4 Norton Orthopaedic Specialists, Louisville, KY, USA; 5 Department of Shoulder and Elbow Surgery, Schulthess Clinic Zurich, Zurich, Switzerland; 6 Department of Shoulder and Elbow Surgery, Charité – Universitaetsmedizin Berlin, Berlin, Germany; 7 St Joseph's Health Care, Hand and Upper Limb Centre, 6221 University of Western Ontario, London, ON, Canada

**Keywords:** Stress shielding, bony adaptations, stemless, anatomic shoulder, shoulder arthroplasty, bone resorption

## Abstract

**Background:**

Finite element analysis has suggested that stemless implants may theoretically decrease stress shielding. The purpose of this study was to assess the radiographic proximal humeral bone adaptations seen following stemless anatomic total shoulder arthroplasty.

**Methods:**

A retrospective review of 152 prospectively followed stemless total shoulder arthroplasty utilizing a single implant design was performed. Anteroposterior and lateral radiographs were reviewed at standard time points. Stress shielding was graded as mild, moderate, and severe. The effect of stress shielding on clinical and functional outcomes was assessed. Also, the influence of subscapularis management on the occurrence of stress shielding was determined.

**Results:**

At 2 years postoperatively, stress shielding was noted in 61 (41%) shoulders. A total of 11 (7%) shoulders demonstrated severe stress shielding with 6 occurring along the medial calcar. There was one instance of greater tuberosity resorption. At the final follow-up, no humeral implants were radiographically loose or migrated. There was no statistically significant difference in clinical and functional outcomes between shoulders with and without stress shielding. Patients undergoing a lesser tuberosity osteotomy had lower rates of stress shielding, which was statistically significant (*p* = 0.021)

**Discussion:**

Stress shielding does occur at higher rates than anticipated following stemless total shoulder arthroplasty, but was not associated with implant migration or failure at 2 years follow-up.

**Level of evidence:**

IV, Case series.

## Introduction

Anatomic total shoulder arthroplasty (TSA) is commonly performed in an effort to reduce pain and improve function by mimicking the native glenohumeral anatomy. Failure following TSA is most commonly related to the glenoid component.^1^ Humeral component failure is rare and is usually related to infection.^2,3^ Traditional longer stem cemented humeral components were problematic in the primary setting as cementation leads to increased operative time, theoretical risk of embolization, and increases the complexity of revision shoulder surgery.^4^ To that effect, many manufacturers transitioned to shorter uncemented humeral implants, including newer stemless canal-sparing prostheses.^3,5–9^ These implants alter stress distribution within the proximal humerus resulting in bony adaptations, which may or may not affect clinical outcomes.^10–15^

Finite element analysis has suggested that stemless metaphyseal fixation more closely mimics the cortical stresses seen in the native humerus and could therefore reduce stress shielding seen with other longer uncemented humeral components.^12^ Bony adaptations in the setting of stemless TSA have been assessed by only a few authors, and predominantly focus on a unique hollow screw design with a trunion.^7,10,14^ The purpose of this study was to assess the radiographic proximal humeral bony adaptations seen following stemless anatomic TSA performed with a press-fit, on-lay, anchor design, and to assess the early influence of stress shielding on clinical and functional outcomes.

## Methods

### Study design

This study was a retrospective review of 152 prospective followed stemless TSAs that were performed as part of the US Food and Drug Administration Investigational Device Exemption study in North America and a European post-market clinical follow-up study. The inclusion and exclusion criteria for the two studies have been previously published.^16,17^ Patients had radiographic studies at each required time point including preoperative, 6 weeks or 3 months, 6 months, 1 year, and 2 years postoperatively. All surgical procedures were performed by 21 experienced shoulder arthroplasty surgeons in the United States, Canada, and Europe using the same implant (Sidus; Zimmer Biomet, Warsaw, IN, USA).

### Surgical technique

A deltopectoral approach was uniformly performed. The subscapularis was managed based on surgeon preference. A lesser tuberosity osteotomy (LTO) was performed in 48 shoulders, a subscapularis peel in 64 shoulders, and a tenotomy in 40 shoulders. An anatomic TSA was then performed in a standard fashion. A standard postoperative rehabilitation protocol was employed beginning with an early passive range of motion (ROM) and a sling for 6 weeks. Full active ROM was initiated at 6 weeks postoperatively with a strengthening program at 12 weeks.

### Outcome measures

Clinical and functional outcomes were assessed at 2 years follow-up utilizing a standardized questionnaire and included ROM, visual analogue scale (VAS) pain, and american shoulder and elbow surgeons (ASES) scores. Patient demographics were also recorded.

**Figure 1.**
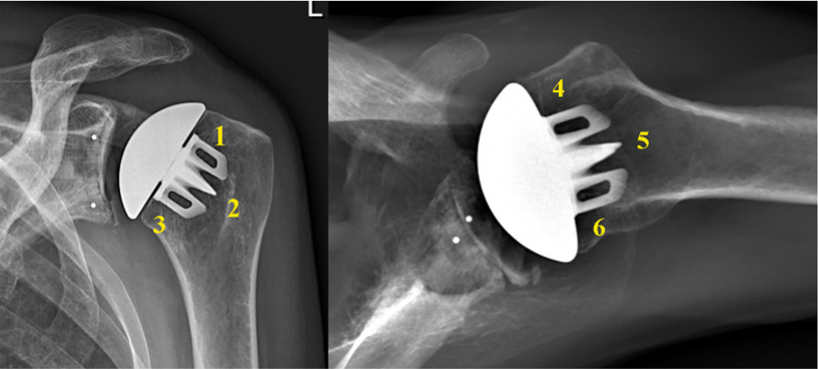
Schematic diagram demonstrating the six zones utilized in this study as proposed by Denard et al.^13^

All radiographs were reviewed by two fellowship-trained shoulder surgeons (WRA, GSA). Anteroposterior (AP) and axillary lateral radiographs were reviewed at all time points. The proximal humerus was divided into six zones as modified by Denard et al. (Figure 1).^13^ Stress shielding was graded as mild for decreased bone density, moderate for cortical thinning, and severe for complete bony resorption. Given that multiple zones may have been involved in each shoulder, grading was classified by the most severe level of stress shielding noted in any single zone. Additionally, humeral implant anchor proximity to the lateral cortex was measured in millimeters. Spot welds were noted. Glenoid component loosening was assessed based on the Lazarus grade.^18^

### Statistical analysis

Continuous data, such as age, patient weight, was summarized using means, standard deviations, minimums, medians, maximums, and 95% confidence intervals. Categorical data, such as sex and approach, were summarized using counts and percentages. Comparisons of demographics, operative information, ROM, ASES, and pain scores between no stress shielding and any stress shielding, and between severe stress shielding at 2 years and less severe stress shielding at 2 years were tested by likelihood ratio tests, score tests, and Wald tests (if separation is absent).

## Results

### Radiographic outcomes

**Figure 2.**
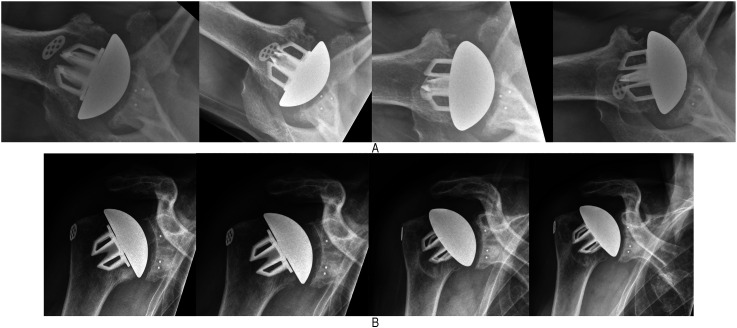
(A) sequential axillary radiographs (L to R: 6 weeks, 6 months, 12 months, and 24 months postoperatively) demonstrating severe stress shielding along the anterior zone. (B) Sequential anteroposterior (AP) radiographs (L to R: 6 weeks, 6 months, 12 months, and 24 months postoperatively) demonstrating moderate stress shielding along the medial calcar and greater tuberosity regions. Note the thinning of the cortex along the medial calcar.

**Figure 3.**
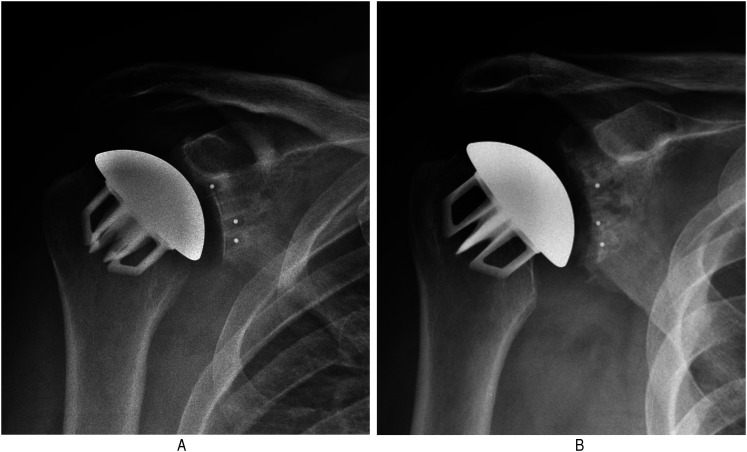
(a) Three-month postoperative anteroposterior (AP) radiograph and (b) 2-year postoperative AP radiograph demonstrating severe stress shielding along the medial calcar.

Bony adaptions were noted in a total of 61 (40%) shoulders (Table 1). Mild adaptation changes were noted in 34 (22%) shoulders. Seventeen (11%) shoulders demonstrated moderate stress shielding (Figure 2B) and another 11 (7%) shoulders demonstrated severe stress shielding (Figure 2A, 3A and 3B).

**Table 1.** Distribution of bony adaptations seen in all shoulders based on zone and severity.

	Mild	Moderate	Severe
Zone 1	12	4	1
Zone 2	4	1	0
Zone 3	24	3	7
Zone 4	14	8	3
Zone 5	3	1	0
Zone 6	10	6	2

In cases of bony adaptation, 34 (57%) shoulders only involved one zone. In these cases, the greater tuberosity was involved in 5 shoulders and the medial calcar in 15. On the lateral radiograph, there was stress shielding anteriorly in 8, posteriorly in 6. Furthermore, the majority of these isolated changes were graded as mild (28 shoulders). Moderate changes were seen in 4 shoulders and severe changes were seen in 2.

Greater tuberosity severe stress shielding was noted in one shoulder. Radiographically, no humeral implants were found to have subsided or migrated. Spot welds were seen in 5 (3%) shoulders. These spot welds were located laterally from the inner aspect of the lateral cortex to the lateral implant fin in all cases.

**Figure 4.**
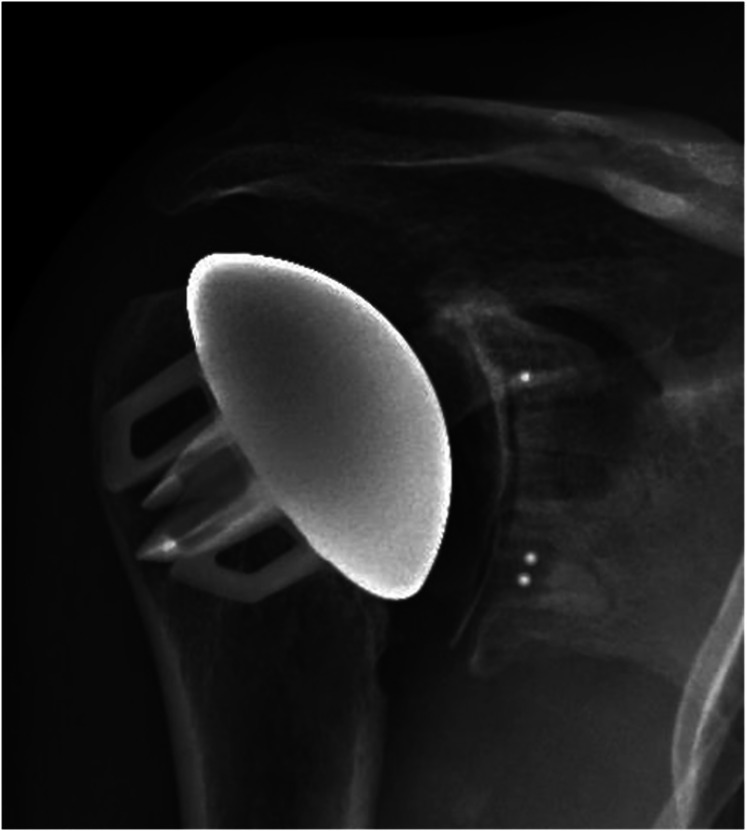
Anchor fins are seen to be in contact with the lateral cortex of the proximal humerus. This is confirmed on multiple views at multiple time points. Complete 2 mm radiolucent lines are observed as well around the glenoid component indicating a potentially loose implant.

The stemless implant was in direct contact with the lateral cortex as visualized on the AP radiograph in 3 (2%) shoulders, while on the lateral radiograph, the anchor fins appeared to contact the endosteum in 19 (13%) shoulders (Figure 4). The average horizontal distance from the lateral cortex to the tip of the anchor fin was 8 mm (range: 0–16 mm). This was not correlated with the presence of stress shielding.

There was a statistically significant difference in the rate of stress shielding based on the subscapularis approach (*p* = 0.021). Stress shielding was noted in 16 (40%) shoulders that underwent a subscapularis tenotomy, 34 (57%) shoulders that underwent a subscapularis peel, and 10 (21%) shoulders that underwent an LTO. The subscapularis peel and tenotomy groups were noted to have a statistically significant increase in stress shielding when compared to the LTO group (*p* < 0.0001 and *p* = 0.017, respectively). There was no significant difference in stress shielding between peel and tenotomy groups (*p* = 0.195). The subscapularis approach, however, did not affect the severity of stress shielding (p = 0.13).

Glenoid radiolucent lines were noted in 28 (18%) patients. These were Lazarus grade 1 in 6, grade 2 in 5, grade 3 in 13, and grade 4 in 2.

### Clinical outcomes

The average forward elevation at 2 years follow-up was 148° ± 26° in shoulders without stress shielding compared to 149° ± 26° in those with stress shielding (*p* = 0.53). The average external rotation at 2 years follow-up was 51° ± 23° in shoulders without stress shielding compared to 52° ± 1° in those with stress shielding (*p* = 0.73). The average VAS pain at 2 years follow-up was 0.8 ± 1.5 in those without stress shielding compared to 0.8 ± 1.8 in those with stress shielding (*p* = 0.88). Similarly, there were no statistically significant differences between any of these parameters when comparing those with severe stress shielding to those with mild or moderate stress shielding (Table 2).

**Table 2.** Comparison of demographic, clinical, and functional outcomes of patients with and without stress shielding, as well as those with severe stress shielding compared to mild or moderate stress shielding.

	No stress shielding	Any stress shielding	*P*-value	Mild or moderate stress shielding	Severe stress shielding	*P*-value
Patient Age (years)	62 ± 8	60 ± 10	0.124	62 ** **± 9	60 ± 5	0.424
Gender (% Female)	46	48	0.738	47	46	0.933
Active forward elevation	148° ± 26°	149° ± 26°	0.936	144° ± 23°	134° ± 41°	0.267
Active external rotation	50° ± 23°	52° ± 17°	0.728	51° ± 21°	53° ± 15°	0.796
VAS	0.8 ± 1.5	0.8 ± 1.8	0.877	0.8 ± 1.6	1.2 ± 2.5	0.435
ASES	89 ± 17	89 ± 16	0.975	90 ± 16	86 ± 18	0.489

### Functional outcomes

The average ASES score at 2 years follow-up was 89 ± 17 in those without stress shielding compared to 89 ± 16 in those with stress shielding (*p* = 0.97). Similarly, there was no statistically significant difference between any of these parameters when comparing those with severe stress shielding to those with mild or moderate stress shielding.

## Discussion

Stemless humeral components have been studied extensively with generally favorable clinical and functional outcomes.^6–9,16,19–21^ These implants have been shown in finite element studies to reduce stress shielding compared to stemmed implants. Additionally, studies have demonstrated that implant design and geometry have an important influence on the bone stresses transmitted to the proximal humerus.^11,12,15^

Habermeyer et al. reviewed 78 patients with 6 years of radiographic follow-up utilizing a hollow screw-type stemless anatomic TSA with a trunnion.^7^ The authors noted decreased cancellous bone density in 46.2% of patients with the majority occurring along the greater tuberosity on the AP and anteriorly on the lateral radiograph. The authors did not grade the amount of stress shielding, nor did they correlate the radiographic findings with clinical outcomes. Studying the same implant, Uschok et al. reported decreased bone density in only 25% of shoulders, which was less than observed with a standard length humeral component by the same manufacturer.^14^ Heuberer et al. reviewed a larger series of patients undergoing stemless TSA and hemiarthroplasty with the same hollow screw design and noted a 37% rate of radiological changes with complete osteolysis in 14% of the hemiarthroplasty cohort and 16% of the TSA cohort.^10^

The rate of stress shielding in our study is similar to that noted in these prior studies utilizing a different type of stemless canal-sparing implant. The predominant location of stress shielding, however, with the anchor fin design assessed in this study was the medial calcar, rather than the superior aspect of the greater tuberosity. This could be related to implant design as suggested by the finite element study performed by Reeves et al.^12^ With the hollow screw-type implant, there is a trunnion that is likely protective for medial calcar bone resorption. The trunnion rests on the humeral head osteotomy surface, so loads are transmitted more uniformly to the underlying bone. As such, the bone in the medial calcar region is not shielded, and therefore does not undergo the same degree of adaptation. Recently, Akilhah et al. compared a hollow screw design to the impaction type design used in this study and demonstrated that the hollow screw with a trunnion may prevent and be protective of medical calcar bone resorption.^22^

Proximal stress shielding may also occur when there is distal contact of the implant with the inner aspect of the lateral cortex. This leads to proximal bone adaptations as the loads are transmitted from the metallic humeral head, thru the stemless implant, and preferentially exit the implant distally at the contact location with the inner cortex. This result in lower stress in the most proximal portions of the humeral bone, and therefore stress shielding with resultant bone adaptations. However, due to the relatively small sample size in this study, we could not demonstrate any correlation between distal spot welds and proximal bone adaptations.

Heuberer et al. demonstrated that radiographic adaptations in the setting of a hollow screw stemless arthroplasty did not affect clinical outcomes based on the Constant score.^10^ This is similar to our study as well, which demonstrated no statistically significant differences in clinical or functional outcomes between shoulders with or without stress shielding. Additionally, when comparing shoulders with severe stress shielding to mild or moderate stress shielding, we found no statistically significant differences in any clinical outcomes.

Our study did demonstrate a substantially lower rate of bony adaptations in patients that underwent a LTO when compared to a subscapularis peel or tenotomy. This may be related to the healing process and bony remodeling of an LTO repair being protective of stress shielding. The subscapularis peel had the highest rate of stress shielding. A potential reason for this is that the peel approach completely detaches all of the musculotendinous attachments of the subscapularis to the lesser tuberosity and the anteromedial calcar region. It is conceivable that incomplete healing occurs, with preferential healing of the upper tendinous portion of the subscapularis, with less predictable healing of the lower muscular portion of the subscapularis to bone. As such, the anteromedial calcar area would be relatively shielded from the normal loads applied by the subscapularis. Although variations were found between approaches in stress shielding, a prior study has demonstrated no difference in clinical outcomes of stemless TSA when comparing the different approaches.^17^ Thus, despite a higher rate of stress shielding with subscapularis peel and tenotomy, there does not appear to be an effect on early clinical outcomes.

The present study does have several strengths. In particular, assessment for stress shielding and bony adaptations was performed by comparing the final radiograph with the early immediate postoperative radiograph at 6 weeks or 3 months. Second, unlike most prior studies, the amount of stress shielding was graded on a mild, moderate, and severe scale to stratify the type of changes. Third, this study involved multiple surgeons in multiple countries and therefore this data can be pragmatically applied to the general orthopaedic surgeon.

Nonetheless, there are also limitations with this study. First, this is a relatively short-term follow-up study with radiographs examined at 2 years postoperatively. Presently, it is unclear whether further follow-up past 2 years would result in delayed onset bone adaptations in patients without stress shielding in the first 2 years. Also, it is difficult to predict if the severity of stress sheidling noted in this study will increase with further follow-up. Additionally, the radiographs used in this study were performed at multiple institutions with different radiographic exposure settings. The effect of this was minimized by utilizing sequential radiographs and comparing immediate postoperative radiographs with those performed at 2-year follow-up. All study sites obtained radiographs in at least three planes (e.g. true AP, axillary, and Y views). Radiographic interpretation of stress shielding was performed on the true AP, or AP with the arm in external rotation to ensure a good greater tuberosity profile. Overall, we suspect that the variable radiographic exposure setting had minimal effect on the ability of the observers to identify bony adaptations.

Stress shielding with this grit-blasted finned stemless on-lay anatomic TSA demonstrates a low rate of significant bone adaptations with no implant failure at early follow-up. This is consistent with early reports of another stemless implant.^7^ Further clinical follow-up and more long-term radiographic assessment are required to determine the natural history of stress shielding in stemless humeral implants.

## Conclusion

Stress shielding does occur with a grit-blasted four-finned stemless humeral implant. The rate of stress shielding in the finned style of implant is similar to that reported in the hollow central screw style stemless implant. Fortunately, the occurrence and severity of bony adaptations had no effect on short-term patient clinical outcomes. Subscapularis management technique did have a substantial effect on the occurrence of stress shielding, with the LTO having the lowest rate and the subscapularis peel technique having the highest rate of bone adaptations.

## References

[1] Matsen FA 3rd Clinton J Lynch J , et al. Glenoid component failure in total shoulder arthroplasty. J Bone Joint Surg Am 2008; 90: 885–896. 18381328 10.2106/JBJS.G.01263

[2] Pottinger P Butler-Wu S Neradilek MB , et al. Prognostic factors for bacterial cultures positive for propionibacterium acnes and other organisms in a large series of revision shoulder arthroplasties performed for stiffness, pain, or loosening. J Bone Joint Surg Am 2012; 94: 2075–2083. 23172325 10.2106/JBJS.K.00861

[3] Aibinder WR Bartels DW Sperling JW , et al. Mid-term radiological results of a cementless short humeral component in anatomical and reverse shoulder arthroplasty. Bone Joint J 2019; 101–B: 610–614. 10.1302/0301-620X.101B5.BJJ-2018-1374.R1 31039055

[4] Harmer L Throckmorton T Sperling JW . Total shoulder arthroplasty: are the humeral components getting shorter? Curr Rev Musculoskelet Med 2016; 9: 17–22. 26801933 10.1007/s12178-016-9313-3 PMC4762805

[5] Athwal GS . Spare the canal: stemless shoulder arthroplasty Is finally here: commentary on an article by R. Sean Churchill, MD, et al.: “clinical and radiographic outcomes of the simpliciti canal-sparing shoulder arthroplasty system. A prospective Two-year multicenter study”. J Bone Joint Surg Am 2016; 98: 28. 10.2106/JBJS.15.01350 27053594

[6] Churchill RS Athwal GS . Stemless shoulder arthroplasty-current results and designs. Curr Rev Musculoskelet Med 2016; 9: 10–16. 26809955 10.1007/s12178-016-9320-4 PMC4762801

[7] Habermeyer P Lichtenberg S Tauber M , et al. Midterm results of stemless shoulder arthroplasty: a prospective study. J Shoulder Elbow Surg 2015; 24: 1463–1472. 25870114 10.1016/j.jse.2015.02.023

[8] Hawi N Magosch P Tauber M , et al. Nine-year outcome after anatomic stemless shoulder prosthesis: clinical and radiologic results. J Shoulder Elbow Surg 2017; 26: 1609–1615. 28410956 10.1016/j.jse.2017.02.017

[9] Huguet D DeClercq G Rio B , et al. Results of a new stemless shoulder prosthesis: radiologic proof of maintained fixation and stability after a minimum of three years’ follow-up. J Shoulder Elbow Surg 2010; 19: 847–852. 20303799 10.1016/j.jse.2009.12.009

[10] Heuberer PR Brandl G Pauzenberger L , et al. Radiological changes do not influence clinical mid-term outcome in stemless humeral head replacements with hollow screw fixation: a prospective radiological and clinical evaluation. BMC Musculoskelet Disord 2018; 19: 28. 29357861 10.1186/s12891-018-1945-6 PMC5778649

[11] Razfar N Reeves JM Langohr DG , et al. Comparison of proximal humeral bone stresses between stemless, short stem, and standard stem length: a finite element analysis. J Shoulder Elbow Surg 2016; 25: 1076–1083. 26810016 10.1016/j.jse.2015.11.011

[12] Reeves JM Langohr GDG Athwal GS , et al. The effect of stemless humeral component fixation feature design on bone stress and strain response: a finite element analysis. J Shoulder Elbow Surg 2018; 27: 2232–2241. 30104100 10.1016/j.jse.2018.06.002

[13] Denard PJ Raiss P Gobezie R , et al. Stress shielding of the humerus in press-fit anatomic shoulder arthroplasty: review and recommendations for evaluation. J Shoulder Elbow Surg 2018; 27: 1139–1147. 29422391 10.1016/j.jse.2017.12.020

[14] Uschok S Magosch P Moe M , et al. Is the stemless humeral head replacement clinically and radiographically a secure equivalent to standard stem humeral head replacement in the long-term follow-up? A prospective randomized trial. J Shoulder Elbow Surg 2017; 26: 225–232. 27856267 10.1016/j.jse.2016.09.001

[15] Favre P Henderson AD . Prediction of stemless humeral implant micromotion during upper limb activities. Clin Biomech (Bristol, Avon) 2016; 36: 46–51. 27236035 10.1016/j.clinbiomech.2016.05.003

[16] Krukenberg A McBirnie J Bartsch S , et al. Sidus stem-free shoulder system for primary osteoarthritis: short-term results of a multicenter study. J Shoulder Elbow Surg 2018; 27: 1483–1490. 29625813 10.1016/j.jse.2018.02.057

[17] Aibinder WR Bicknell RT Bartsch S , et al. Subscapularis management in stemless total shoulder arthroplasty: tenotomy versus peel versus lesser tuberosity osteotomy. J Shoulder Elbow Surg 2019; 28: 1942–1947. 31078408 10.1016/j.jse.2019.02.022

[18] Lazarus MD Jensen KL Southworth C , et al. The radiographic evaluation of keeled and pegged glenoid component insertion. J Bone Joint Surg Am 2002; 84: 1174–1182. 12107318 10.2106/00004623-200207000-00013

[19] Beck S Beck V Wegner A , et al. Long-term survivorship of stemless anatomical shoulder replacement. Int Orthop 2018; 42: 1327–1330. 29368045 10.1007/s00264-018-3779-0

[20] Churchill RS Chuinard C Wiater JM , et al. Clinical and radiographic outcomes of the simpliciti canal-sparing shoulder arthroplasty system: a prospective Two-year multicenter study. J Bone Joint Surg Am 2016; 98: 552–560. 27053583 10.2106/JBJS.15.00181

[21] Athwal GS Krupp RJ Carlson G , et al. A multicenter, prospective 2-year analysis of the sidus stem-free shoulder arthroplasty system. JSES Int 2020; 4: 120–126. 32544936 10.1016/j.jses.2019.10.005 PMC7075750

[22] Alikhah A Imiolczyk JP Krukenberg A , et al. Screw fixation in stemless shoulder arthroplasty for the treatment of primary osteoarthritis leads to less osteolysis when compared to impaction fixation. BMC Musculoskelet Disord 2020; 21: 95. 32398035 10.1186/s12891-020-03277-3 PMC7218655

